# Immediate versus sustained effects: interrupted time series analysis of a tailored intervention

**DOI:** 10.1186/1748-5908-8-130

**Published:** 2013-11-05

**Authors:** Andria Hanbury, Katherine Farley, Carl Thompson, Paul M Wilson, Duncan Chambers, Heather Holmes

**Affiliations:** 1 Department of Health Sciences, University of York, Heslington, York YO10 5DD, UK; 2 Centre for Reviews and Dissemination, University of York, Heslington, York YO10 5DD, UK; 3 West and South Yorkshire Bassetlaw Commissioning Support Unit, Douglas Mill, Bowling Old Lane, Bradford BD5 7RJ, UK

**Keywords:** Time series, Process evaluation, Sustainability, Educational meetings, Reminders, Educational materials

## Abstract

**Background:**

Detailed intervention descriptions and robust evaluations that test intervention impact—and explore reasons for impact—are an essential part of progressing implementation science. Time series designs enable the impact and sustainability of intervention effects to be tested. When combined with time series designs, qualitative methods can provide insight into intervention effectiveness and help identify areas for improvement for future interventions. This paper describes the development, delivery, and evaluation of a tailored intervention designed to increase primary health care professionals’ adoption of a national recommendation that women with mild to moderate postnatal depression (PND) are referred for psychological therapy as a first stage treatment.

**Methods:**

Three factors influencing referral for psychological treatment were targeted using three related intervention components: a tailored educational meeting, a tailored educational leaflet, and changes to an electronic system data template used by health professionals during consultations for PND. Evaluation comprised time series analysis of monthly audit data on percentage referral rates and monthly first prescription rates for anti-depressants. Interviews were conducted with a sample of health professionals to explore their perceptions of the intervention components and to identify possible factors influencing intervention effectiveness.

**Results:**

The intervention was associated with a significant, immediate, positive effect upon percentage referral rates for psychological treatments. This effect was not sustained over the ten month follow-on period. Monthly rates of anti-depressant prescriptions remained consistently high after the intervention. Qualitative interview findings suggest key messages received from the intervention concerned what appropriate antidepressant prescribing is, suggesting this to underlie the lack of impact upon prescribing rates. However, an understanding that psychological treatment can have long-term benefits was also cited. Barriers to referral identified before intervention were cited again after the intervention, suggesting the intervention had not successfully tackled the barriers targeted.

**Conclusion:**

A time series design allowed the initial and sustained impact of our intervention to be tested. Combined with qualitative interviews, this provided insight into intervention effectiveness. Future research should test factors influencing intervention sustainability, and promote adoption of the targeted behavior and dis-adoption of competing behaviors where appropriate.

## Background

Tailored implementation strategies require developers to choose an underpinning theory, constructs, or framework as a guide to exploring the barriers to performing the target behavior, and select intervention strategies tailored to these. This process is complex, with multiple decisions to be made by developers at each stage. Consequently, detailed reporting of intervention design and delivery [1], and robust evaluation focussing not only on the impact of the intervention, but also on the underlying reasons for the impact [2] is required.

Interrupted time series designs (ITS) compare multiple before and after measures to detect whether an intervention has had an impact greater than any underlying trend in the data [3], and have been recommended for evaluating intervention effectiveness [4]. In controlling for underlying trend, time series designs are particularly suitable to implementation studies in which innovation adoption may increase over time [5,6], independent of the effects of an intervention. Time series designs also enable duration of intervention effect to be studied. While the sustainability of interventions is gaining research attention [7], it is rarely reported on [8,9]. Being based largely on routinely collected health services data, time series design overcome some of the shortcomings of self-report questionnaire-based measures: difficulty in achieving satisfactory response rates [10], and social desirability bias that overestimates intervention effectiveness [11]. On its own, however, a time series design cannot help explain why an intervention has worked (or not). Qualitative process evaluation can help explore the mechanisms underpinning intervention effectiveness and explain an interventions effectiveness [2]. A combination of both approaches promotes a fuller picture on intervention effectiveness.

This article summarizes the development, delivery, and evaluation of a tailored intervention targeting primary care health professionals’ adoption of a National Institute for Health and Clinical Excellence recommendation [12] that women with mild to moderate postnatal depression (PND) should be referred for psychological therapy as a first stage treatment.

## Method

### Design, setting, participants, and measures

The research was conducted in one Primary Care Trust (PCT) in the north of England, UK, with approval granted by the Local Research Ethics Committee (09/H1311/81). All general practitioners (GPs) (n = 495) and nurse practitioners (n = 16) were targeted due to their contact with new mothers, and their role as primary referrers for psychological treatments. Only GPs can routinely prescribe antidepressants, but some appropriately trained nurse practitioners can also prescribe. We used an embedded mixed methods design [13], comprising interrupted time series and qualitative interviews, enabling the measurement and contextual interpretation of intervention impact. Quantitative and qualitative data were collected and analyzed separately, but concurrently, and the point of interface was at interpretation [13].

### Procedure

#### Intervention development

Following a questionnaire and interview-based diagnostic analysis [10], and guided by an underpinning framework [14], the intervention was designed to target influential factors affecting referrals for psychological treatments.

Factors identified were:

1. Awareness of and familiarity with the national recommendations, including awareness of the range of psychological treatments and their availability locally.

2. Awareness and recognition of local expertise in the psychological treatment of PND.

3. Skills to manage patient expectations of treatment (*e.g.*, that they will be immediately prescribed an anti-depressant).

For tailored intervention development, there is a currently a lack of conclusive evidence on the effectiveness of different interventions to target different barriers [15]. Michie *et al*. [16] have proposed a ‘behavior change wheel’ as a means of linking barriers (summarized as ‘capability’: capacity to perform the behavior, including skills and knowledge; ‘opportunity’: factors outside control of the individual that make behavior possible/prompt it; ‘motivation’: goals, decision making), to ‘intervention functions’ (*e.g.*, ‘education,’ ‘persuasion’). Mapping our factors onto Michie *et al*.’s categories of barrier, factors one and two would most closely match the ‘capacity’ barrier, which in turn is linked to education, training, and enablement intervention functions, and thus amenable to targeting via educational materials/meetings/outreach. Factor three would map most closely onto the ‘opportunity’ barrier, linked to ‘environmental restructuring,’ ‘restriction; and ‘enablement’ intervention functions; for example, using reminder systems. The PCT quality improvement team were consulted to assess planned intervention acceptability and feasibility. Three intervention components were then developed (details regarding content and delivery are provided in Additional file 1).

### Component one: tailored educational materials

This was a four-page document providing a summary of the range of, and evidence for, locally available treatments, including self-help and group-based interventions (key factor 1). Educational materials are suited to targeting health professionals’ knowledge and skills gaps [15]. While associated with only small effects on professional practice [17], educational materials are relatively low cost and feasible in most settings, making them a potentially worthwhile intervention [18].

### Component two: tailored educational meeting

This meeting provided an overview of locally available psychological treatments, their evidence-base, local expertise to deliver it (factors one and two), and demonstrated techniques in managing patient expectations (factor three). Educational meetings can result in small improvements in professional practice [19]. Didactic meetings are suited to targeting knowledge-related barriers (factors one and two), and interactive meetings to targeting skills (factor three) [15]. Our educational meeting included both didactic and interactive components, and emphasized the potentially serious nature of PND, based on the finding that impact may be smaller for outcomes perceived as having less serious consequences for patients, and may be greater for mixed interactive and didactic sessions [19]. Educational meetings are considered feasible in most settings, making them a commonly used strategy [15].

### Component three: reminder system

Patient records are recorded electronically during consultations via templates that use specific codes to record diagnoses and treatment choices and provide prompted clinical actions for specific clinical conditions. Two changes were made to the depression template used locally by all GPs and nurse practitioners; these targeted factor one by adding a tab onto the template summarizing the recommendation and locally available psychological treatments. Reminder systems are associated with small to moderate effects [20]. Using the template enabled us to target all relevant health professionals, including those who did not attend the educational event and who may not have accessed the educational materials. No other modifications were introduced to the system during the intervention/post-intervention period.

### Analysis

#### Interrupted time series analysis

Routinely collected data for all women with a depression diagnosis recorded within 12 months of giving birth were downloaded from the electronic recording system used in the PCT, spanning July 2007 to January 2012, providing 45 pre- and 10 post-intervention data points. Data are entered onto the system by all GPs and nurse practitioners. For identified cases, referrals to any psychological treatment, and first time prescriptions (to avoid repeat prescriptions) for any anti-depressants recorded within 18 months of the diagnosis were downloaded. We specified 18 months to ensure those diagnosed towards the end of the first year of giving birth had opportunity to receive a referral or treatment. The inclusion criteria were developed in consultation with the local data quality team to ensure all possible cases were identified. To enable time series analysis, the aggregated data were disaggregated into monthly percentage referral rates for psychological treatments (monthly number of referrals divided by monthly number of cases) and anti-depressant prescribing rates (monthly number of first prescriptions divided by monthly number of cases).

Sequence charts of monthly numbers of PND cases, percentage referral rates for psychological treatments, and percentage first prescription rates for anti-depressants were plotted to examine for any increases over time. For both outcome measures, autocorrelation (ACF) and partial autocorrelation (PACF) plots for the pre-intervention only data were examined to identify the best fitting model using autoregressive integrated moving average (ARIMA) modelling; with ARIMA and time series regression models suggested for analysing such data due to their ability to identify potential biases in the data [4]. Ljung box Q fit statistic and residual plots were examined to assess the extent to which the model successfully removed trend and autocorrelation from the data. The identified models were applied to the entire data series, with the intervention modelled as an immediate and temporary effect spanning April to June 2011, and an immediate and permanent effect from April 2011 onwards.

#### Qualitative interviews

Interviews were conducted in September and October 2011, after delivery of the components, but before too long had passed, hampering recall. Sampling was purposive, with participants randomly, selected to minimize bias, from those participating in the educational meeting to ensure that, theoretically, participants had been exposed to all intervention components. This was because we were interested in the experiences of all intervention components, and so we chose to exclude clinicians who may have been completely unaware of the intervention. From the list of those who attended the educational meeting, invitees were randomly selected, after which, to increase recruitment, all attendees of the event were targeted by telephone. Eight GPs agreed to participate. All interviews took place in participants’ own offices and were digitally recorded and transcribed. Three interviewers were used who had not been directly involved in the delivery of the education events themselves. Respondents were informed that the research was evaluative, and interviews lasted twenty minutes on average.

Interviews were semi-structured (see Additional file 2 for schedule). Data were analyzed using thematic analysis comprising: data familiarization, identification of a thematic framework, and indexing, mapping, and interpretation with reference to the overall aim of the study as well as the themes revealed by the data [21]. One researcher (KF) identified initial themes and subcategories, a second researcher (AH) coded individual transcripts and both coded one identical transcript to assess consistency of interpretation.

## Results

### Exposure to intervention components

Table 1 summarizes the number of GPs and nurse practitioners estimated to have been reached by each intervention component. All GPs and nurse practitioners should have received an electronic and/or paper based copy of the educational materials, and had access to the template changes embedded within the electronic recording system. The educational session, on the other hand, required active attendance.

**Table 1:** Summary of intervention components and estimated numbers receiving

**Intervention component**	**Numbers estimated as receiving intervention component**
Educational materials	Electronic and hard copies sent to all general practitioners (N = 495) and nurse practitioners (N = 16)
Educational event	Total attendance: N = 110 (General practitioners: N = 44; Nurse practitioners: N = 0, ‘Other staff not explicitly targeted but welcome to attend’: N = 66)
Reminder system	Accessible to all GPs (N = 495) and nurse practitioners (N = 16)

### Time series analysis

#### PND cases

A total of 35,436 live births were recorded were recorded during the period July 2007 to January 2012, with 1,849 of these having a diagnosis of PND recorded (overall average of 5.22% of cases). The mean monthly number of recorded PND cases was 33. This, however, increased during the study period, with an average of 30 cases a month (SD 4.13) in 2008 towards the start of the series, and an average of 40 (S.D 6.48) in 2011 toward the end of the series, suggesting more women seeking help, or greater awareness among health professionals over time.

### Referral rates for psychological treatments

**Figure 1:**
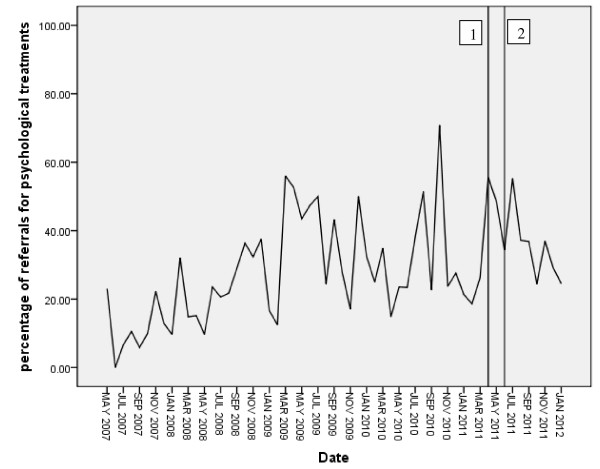
Time series plot of percentage of PND cases receiving referral for psychological treatment.

Figure 1 summarizes monthly percentage referral rates for women with mild to moderate PND, with the timing of intervention components indicated. Of the 1,849 recorded cases of PND in the data series, 550 received a referral for psychological treatment (29.75% of cases). On average, 25% of PND cases received a referral in the six months prior to the intervention, and 45% six months after the intervention was delivered.

The series was differenced due to the upward trend in the data. The ACF and PACF plots suggest a mixed autoregressive and moving average model. An initial ARIMA 111 model was specified. The Ljung box Q fit statistic was non-significant, and the ACF and PACF plots indicated that the non-random variation was removed. However, the autoregressive lag was not significant, while the moving average lag was, suggesting a moving average model would be better. Therefore, a 011 model was fitted.

The 011 model was applied to the full series, with an outlier (October 2010) identified during baseline modelling replaced with the mean of nearby points. The model was run with the intervention fit as an immediate onset short-term effect, followed by an immediate onset, permanent, effect. The short effect model indicated a significant intervention, β = 11.13, t = 2.14, p ≤ 0.05. The permanent effect model found the intervention to be non-significant (β = −0.378, t = −0.241, p = 0.811). Fit statistics are summarized in Table 2.

**Table 2:** Summary of fit statistics for referral to psychological treatments time series model

**Model**	**Adjusted R**^**2**^	**Mean absolute percentage error**	**Ljung-box**	**Auto correlation function plot**	**Partial autocorrelation function plot**	**Significance of intervention**	**Bayesian information criteria**
**Baseline**	0.63	36.17	p ≥ 0.05	No significant lags	No significant lags	-	5.03
**Intervention: short effect**	0.34	35.18	p ≥ 0.05	No significant lags	No significant lags	p ≤ 0.05	5.20
**Intervention: permanent effect**	0.27	35.85	p ≥ 0.05	No significant lags	Significant at lag 18	p ≥ 0.05	5.19

### Prescribing rates for anti-depressants

**Figure 2:**
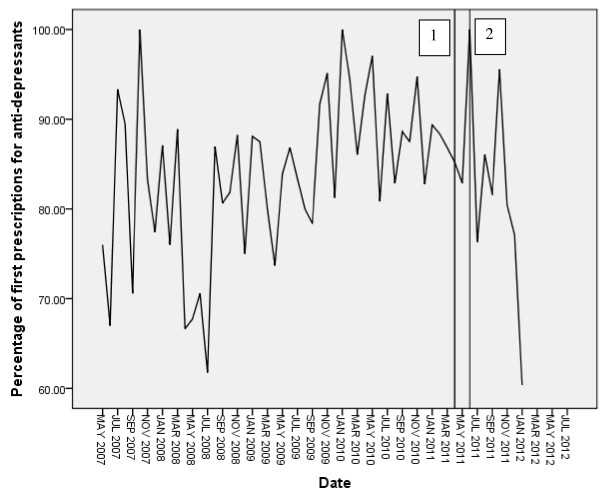
Time series plot of percentage of PND cases receiving first prescription for antidepressants.

Figure 2 summarizes monthly first prescription rates for anti-depressants. Of the 1,849 cases of PND recorded during the entire data series, 1,559 received a first prescription for antidepressants (84.32%). Rates remain high throughout the series; average prescribing rates in the six months before the intervention were 88.26%, and in the six months post-intervention were 85.33%. The ACF and PACF plots suggest a mixed model; therefore, an ARIMA 101 model was initially specified. The Ljung box Q fit statistic and ACF and PACF plots indicate the model to have removed all significant non-random variation from the data series, and both the autoregressive and moving average lags were significant.

The 101 model was applied to the full data series, with the intervention modelled as an immediate onset short and then permanent effect. The intervention was non-significant for both the short (β = −0.004, t = −0.78, p = 0.938) and permanent (β = −0.019, t = − 0.691, p = 0.493) models. Fit statistics are summarized in Table 3.

**Table 3:** Summary of fit statistics for first antidepressants prescriptions time series model

**Model**	**Adjusted R**^**2**^	**Mean absolute percentage error**	**Ljung-box**	**Autocorrelation function plot**	**Partial autocorrelation function plot**	**Significance of intervention**	**Bayesian information criteria**
**Baseline**	0.84	1.95	p ≥ 0.05	No significant lags	No significant lags	-	−5.87
**Intervention: short effect**	0.84	1.95	p ≥ 0.05	No significant lags	No significant lags	p ≥ 0.05	−5.78
**Intervention: permanent effect**	0.94	1.92	p ≥ 0.05	No significant lags	No significant lags	p ≥ 0.05	−5.79

### Qualitative findings

Two key themes emerged:

### Remaining barriers to the use of Psychological Therapies

Many of the barriers discussed reflected those identified pre-intervention [10]. Patient preference for antidepressants, language difficulties (accessing a non-English counsellor), and practical factors (access to childcare, time required), were all cited. This suggests that these barriers were still perceived to be an issue, even though all three intervention components promoted the range of psychological treatments available locally, and that some are easier to access. Concerns about waiting lists for access to psychological treatments remain, suggesting these are still seen predominantly as meaning treatments provided one to one by psychologists:

‘It doesn’t necessarily mean that if you make the appointment for them that they’re going to keep it either, [because] I don’t think that system worked terribly well either. Sometimes if, if, if it’s going to be too long ahead before people get appointments then that can be off-putting for people.’ (R1).

‘But some people, they want a pill to take and they don’t want to go, because to them the time involved is more, isn’t it, having to go for psychological therapy than it is in coming to the doctor and being given a prescription and taking pills.’ (R1).

Medication remains at the forefront of respondents’ minds when asked about treatments. The session on prescribing was a significant part of the messages received at the educational meeting and may explain the lack of significant change in prescribing behavior in the time series analysis:

‘[I thought the]…view of the drug treatment in pregnancy and breast feeding I thought was very helpful as a clinician, and I found the bits after that less’ (R3).

This is supported by one respondent mentioning both an increase in use of referrals for psychological therapies and improvements in appropriate prescribing (Fluoxetine: the anti-depressant recommended for the postnatal period at the educational event). This suggests that while anti-depressant prescribing remains high post-intervention, there may have been a positive impact on GPs prescribing the most appropriate antidepressants to treat PND. There was, however, some confusion about the role of psychological therapies merely as a stop-gap until antidepressants take effect or whether antidepressants served this role until psychological therapies were available.

### Intervention impact on practice and perceptions

The perceived impact of the educational meeting on practice was mixed but generally positive. This included a stated greater willingness or preference for psychological treatments than prior to the educational meeting and a lower threshold of referring earlier. A specific skill put into practice included mini-cognitive behavioral therapy (CBT), covered at the educational meeting:

‘ …and I have done the course, ten minute CBT course as well, it was, I thought that was very, very helpful and I have used it since with some patients.’ (R3).

It was also clear that participants’ practice was affected by learning (at the educational meeting) that GPs can request that women with PND are seen more quickly than some other patients. The recognition of psychological therapies as a long-term investment (‘future-proofing’) was apparent in the descriptions of several respondents and seemed to play a key role in their understandings. Understandings of the benefits of psychological treatments included long term effects, and impact on children in vulnerable/deprived areas.

## Discussion

We delivered a multifaceted, tailored, intervention aimed at increasing referrals of women with mild to moderate PND for psychological treatments as a first stage treatment. Our interrupted time series analysis on monthly referral for psychological treatments data suggests the impact of our intervention on referrals had a short-term 11% effect that was not sustained over the ten-month post-intervention period. An additional interrupted time series analysis run on antidepressant prescribing rates also indicated no impact, with rates remaining high post-intervention.

The finding that the increase in referrals for psychological treatments was not sustained was disappointing. The intervention was multifaceted to try and ensure that all three factors identified from our diagnostic analysis were targeted, and to increase the likelihood of the health professionals being exposed to at least one of the components, with an appreciation that not all health professionals would engage with all components. In example, while the educational meeting, targeting all three key factors, was run twice to facilitate attendance, only 44 GPs attended. The educational materials, targeting factors one and two, were distributed as both electronic and hard copies, but still required recipients to engage with the content. The reminder system, targeting factor one, whereas, was embedded within the electronic recording system used by all GPs and nurse practitioners and was anticipated to produce more lasting effects than the other components due to this. There is currently, however, a lack of sound evidence available to guide the selection of intervention components to target specific barriers [15], let alone to predict duration of effect. For example, it is possible that the impact of the intervention could have been more sustained had an entirely different type of component been included (for example, opinion leaders), with some evidence to suggest that educational meetings-as a single component- may not be effective for changing more complex behaviors [19].

The time series analysis run on the percentage of first drug prescriptions found no significant effect on anti-depressant prescribing, with rates remaining high post-intervention. Implementation research typically seeks to increase adoption of innovations. However, in order to increase the adoption of certain innovations, in some instances other competing clinical actions need to be dis-adopted [22]. We aimed to increase adoption of the recommendation that women with mild to moderate PND should receive referral for psychological treatments. Our three intervention components promoted adoption through targeting influential factors identified from our diagnostic analysis. Our implementation strategy might have been more effective, however, if it had also directly challenged the health professionals’ attitudes towards anti-depressant prescribing. Future implementation studies may benefit from a more holistic approach, promoting adoption of new, targeted, innovations and dis-adoption of competing clinical actions where appropriate.

### Limitations

A limitation of the study is the small number of PND cases diagnosed each month; this averaged 33 but increased over time, suggesting a potential increase in awareness of PND amongst either patients or the health professionals. There is, however, no formal guidance regarding the appropriate number of cases making up each data point in a time series analysis. The number of cases each month, however, may have made the analysis more vulnerable to exaggerated peaks and troughs in the data that were difficult to model robustly: the mean percentage of model error reported in the model fit statistics was high. We considered combining the data into two monthly intervals, but this would have resulted in loss of data points, particularly in the post-intervention period. We also recognize that the number of health professionals interviewed for the qualitative element is small, but it was not intended to provide a second outcome measure to corroborate the ITS analysis. Rather, we sought to provide some explanation for the outcomes identified. Further rich exploration and analysis would be needed to fully understand the way in which messages have been received, understood and applied.

A mediational analysis would have enabled a more precise understanding of intervention impact, comparing pre-and post-intervention survey measures across each of the targeted factors. This had been planned; however, a particularly low response rate for the baseline survey [10] would have made any analyses underpowered, and so the pre-intervention questionnaire was not re-administered post-intervention to avoid wasting health professionals’ time completing a survey that we could not analyze robustly. In not re-administering the survey, we, however, also lost the opportunity to collect self-report fidelity measures that had been designed to be added into the survey, and asked about extent of engagement with the intervention to enable subgroup analyses at the health professional level by degree of engagement. An attempt was, instead, made to obtain fidelity measures for health professionals’ receipt of the educational materials and their accessing of the template changes. This was through the system used in the host site to distribute electronic documents, and through the electronic recording system that the template/reminder system was embedded in, with an interest in identifying those health professionals who had engaged with these two components. However, limitations of the two systems meant that this was not possible. Similarly, to substantiate the finding from the qualitative interviews that the intervention may have resulted in more appropriate prescribing of anti-depressants post-compared with pre-intervention, data were downloaded on the types of drugs prescribed to enable a statistical test of difference in prescribing patterns. However, system limitations again prevented this, with the searches run on the local electronic recording system requiring overly complex combinations of ‘if, then’ rules that resulted in the returned data lacking robustness. Overall, the lack of formal fidelity measures, beyond attendance at the educational event, and the aggregated nature of our two time series outcome measures meant we are unable to reliably assess the extent to which the intervention was delivered as planned, and whether this impacted on intervention effectiveness. Future research, therefore, should build in fidelity measures at the outset, to enable these relationships to be explored.

Our findings do, however, illustrate the importance of using multiple outcome measures. If we had only analyzed referrals data, the lack of impact on prescribing rates, and professionals’ simultaneous referring and prescribing behavior would not have emerged. Although limited in number, the interviews provide some insight into intervention effectiveness, suggesting that the lack of change in prescribing behavior may be due to mixed messages received from the intervention. For example, it is possible that our attempts to ensure that our messages were balanced with recognition of the need for stepped care led to some participants focussing on mixed psychological and anti-depressants treatments, and some taking home a message reinforcing use of anti-depressants. The interviews also provide some suggestion that the intervention messages may not have been adequately targeted by our intervention; with some of the barriers cited mirroring those identified through our diagnostic analysis.

## Conclusions

This paper summarizes the development, delivery, and evaluation of a tailored intervention targeting compliance with nationally recommended psychological treatments for mild to moderate PND. An interrupted time series design using two outcome measures established that any impact was short-term and applied only to the behavior we had directly targeted—referrals for psychological treatment—rather than the related behavior- anti depressant prescription rates. Use of qualitative interviews provided some insight into why the intervention had not had a significant and sustained impact. Future research should continue to use multiple outcome measures, focus attention on intervention sustainability, and empirically test the impact of targeting both adoption and disadoption.

## Abbreviations

ACF: Autocorrelation function plot; ARIMA: Autoregressive integrated moving average; CBT: Cognitive behavioral therapy; GPs: General practitioners; ITS: Interrupted time series; PACF: Partial autocorrelation function plot; PCT: Primary care trust; PND: Postnatal depression.

## Competing interests

PMW is an editor for Implementation Science and was not involved in review of this manuscript. All other authors declare that they have no competing interests.

## Authors’ contributions

AH devised the time series design and ran the time series analyses, developed the intervention and drafted the manuscript. KF advised on the qualitative interviews and analyzed the data, developed the intervention and contributed to the manuscript. CT contributed to development of intervention, and contributed to the manuscript. PW contributed to development of the intervention, and commented on the manuscript. DC contributed to development of the intervention, and commented on the manuscript. HH prepared the datasets for analysis, contributed to qualitative data collection and contributed to the manuscript. All authors read and approved the final manuscript.

## Supplementary Material

Additional file 1 Summary of intervention components, content and delivery. Click here for file

Additional file 2 Interview schedule. Click here for file
