# Supplementary material for: Immediate versus sustained effects: interrupted time series analysis of a tailored intervention
Source: Implement Sci. 2013 Nov 5;8:130. doi: 10.1186/1748-5908-8-130 (PMC4228338; doi:10.1186/1748-5908-8-130)
Supplement: Additional file 1 — Summary of intervention components, content and delivery. [file 1748-5908-8-130-S1.pdf]

Additional file 1 Summary of intervention components, content and delivery

Component and delivery	Factors targeted and content
**Educational materials (component 1)**<br><br>Delivered April 2011 as a link embedded within an email. Hard copies also sent to practice managers, for their distribution to practice staff, 2 weeks later. The Medical Director provided the email signatory for electronic and paper versions. No fidelity measure was collected to assess the number of health professionals who accessed the electronic or paper based versions of the document.	**Factor 1**<br><br>Page 1: bullet point summary of document covering proportion of women affected by postnatal depression nationally, and potential high risk in local population; NICE recommendation for psychological therapies as first step treatment; effectiveness of these; promotion of intervention component three (see below)<br><br>Page 2: National prioritisation of maternal mental health and impact on children; how to identify cases; summary of psychological therapies<br><br>Page 3: Given range of treatments available locally, some can be started immediately as they do not have a waiting list; effectiveness of the different therapies; role of anti-depressants<br><br>Page 4: Summary table of therapies, their effectiveness, quality of evidence, and availability locally
**Educational meeting (component 2)**<br><br>Delivered in June 2011 by local health professionals with expertise in the clinical management of postnatal depression. The same event was run twice; once as a day time and once as an evening event. Promotion was through, 1) email text used to send the tailored educational materials	**Factors 1, 2, 3**<br><br>Session 1: Introduction and promotion of forthcoming changes to the depression recording template through use of screen shots in a PowerPoint presentation (component 3, below, delivered by Data Quality Manager)

(April), 2) advertisement in two electronic staff newsletters emailed across the PCT (one specifically targeting GP practices, May); 3) email sent by a member of the host sites' quality improvement team one week before the event. Event attendance was recorded.	Session 2: Proportion of women with postnatal depression; NICE recommendations for identification; NICE recommendations for stepped care and the range of psychological treatment options (PowerPoint presentation delivered by local GP/Mental Health Lead)<br><br>Session 3: CBT for a ten minute consultation (including practical session); self-help for those who can self-help (PowerPoint presentation and small-group interactive session delivered by local GP/Mental Health Lead)<br><br>Session 4: Role and expertise of the primary care mental health team; potential severity of PND and impact on children; how to refer, including fast track referral for postnatal depression; assessment and treatments available; tackling of access issues through availability of flexible appointment slots, interpreters, children's centre venues, accessible leaflets and self-help books (PowerPoint presentation delivered by two members of the PCMHT; both were primary care counsellors and one was also the maternal mental health lead).<br><br>Session 5: Patient experience of postnatal depression, to illustrate impact on women and their families (delivered by a patient with experience of PND who discussed her experience)
**Reminder system (component 3)**<br><br>Two changes were introduced in June 2011 to the depression template used locally by all GPs and nurse practitioners. Promotion was through,	**Factor 1**<br><br>Change one: prompt asking whether patient is a female who has given birth in the past 12 months, with tabs added linking to

Additional file 1 Summary of intervention components, content and delivery

1) the educational materials distributed in April 2011, 2) the educational event in June 2011. All general practitioners and nurse practitioners have access to the system; however, system limitations meant we were unable to monitor the number of hits the template received before compared with after the introduction of the changes.	NICE recommended screening tools, including Edinburgh Postnatal Depression Scale<br><br>Change two: 'treatment options for postnatal depression' tab added in, summarised by mild, moderate and severe postnatal depression and covering the range of locally available treatments, including guided self-help, listening visits, cognitive behavioural therapy
