# Supplementary material for: Immediate versus sustained effects: interrupted time series analysis of a tailored intervention
Source: Implement Sci. 2013 Nov 5;8:130. doi: 10.1186/1748-5908-8-130 (PMC4228338; doi:10.1186/1748-5908-8-130)
Supplement: Additional file 2 — Interview schedule. [file 1748-5908-8-130-S2.pdf]

Additional file 2: interview schedule

This year, the trust conducted quality improvement work in the clinical area of postnatal depression. This involved the introduction of a modified SystemOne template for postnatal depression; distribution of an evidence-briefing leaflet highlighting the range of and evidence base for locally available psychological therapies, and two educational events run in June

1. Before the quality improvement work, how familiar would you say you were at the time with treatment choices for mild to moderate postnatal depression? (Prompts: were you aware of the guidance? Which treatment choices were you familiar with?)

2. Has the quality improvement work had any impact on either your practice or planned practice regarding treatment choices for women with mild to moderate postnatal depression?

   If yes,

   - What and to what extent?
   - Thinking of the modified SystemOne template, the TRiPLaB evidence-briefing leaflet, the short leaflet and the educational event, which do you think was most influential?
   - Why do you feel this is the case?

   If not,

   - Why do you feel this is?

3. What do you think of the evidence base for psychological treatments for postnatal depression? (Prompt: how strong do you think that it is?)

    - Can you explain why you think this?

4. What do you think about the cost effective of psychological treatments for postnatal depression? (Prompt: how cost effective do you think they are?)

    - Can you explain why you think this?

5. What do you feel you learnt from the educational event?

6. Since the educational event, do you feel that you have developed any new contacts, either within or outside your own practice, who you feel you can talk to about the clinical management of postnatal depression? (Prompt: for example, if you felt unsure about something and needed advice)

7. Since the educational event, have you discussed the content of it with any colleagues or other members of your practice?

8. Do you feel that there are any barriers or obstacles to you making referrals for psychological treatments for women with mild to moderate postnatal depression?

    If so:

    - What are they?
    - Do you feel there has been any **reduction or improvement in these barriers/obstacles** as a consequence of the quality improvement work (the modified SystemOne template, the TriPLaB evidence-briefing leaflet, the short leaflet and the educational event)?

9. Thinking of the modified SystemOne template, the TriPLaB evidence-briefing leaflet, the short leaflet and the educational event, are there any changes that you would make to help improve them? (Prompt: any changes to the template? Any changes to the evidence-briefing leaflet? Any changes to the short leaflet? Any changes to the educational event?)

10. Can you suggest any further changes that could be made to help health professionals make best use of the psychological therapies available in the Trust?

11. Are you aware of any other activities in the Trust that support staff in making referrals for psychological treatments for postnatal depression? (Prompt: any other training, for example)

    If so

    - What? Do you consider them to have been effective?

Thank you for your time.
